# Intestinal obstruction: a rare complication of channeling Transurethral Resection of the Prostate (TURP): a case report

**DOI:** 10.1186/1752-1947-2-30

**Published:** 2008-01-29

**Authors:** AA Popoola, KA Onawola, MD Adesina, IO Olaoye

**Affiliations:** 1 Urology Division, Department of Surgery, University of Ilorin Teaching Hospital, Ilorin, Nigeria; 2 General Surgery Division, Department of Surgery, University of Ilorin Teaching Hospital, Ilorin, Nigeria

## Abstract

**Introduction:**

Channeling transurethral resection of the prostate is a recognized form of adjunctive treatment in the treatment of patients with prostate cancer. Despite the fact that complications arising from the procedure have been on the decline, rare complications like intestinal obstruction may occur.

**Case presentation:**

This is a case report of a 56 year old man who developed mechanical intestinal obstruction few days after a channeling TURP for advanced CaP.

**Conclusion:**

The report highlights the possibility of intestinal obstruction as a secondary event following a silent urinary bladder perforation during channeling TURP. Early recognition and intervention were responsible for the good outcome in this patient.

## Introduction

Transurethral resection of the prostate (TURP) represents the accepted standard of surgical therapy for the management of symptomatic bladder outlet obstruction due to benign prostatic hyperplasia (BPH) [1]. Limited or Channeling TURP is also a recognized form of adjunctive treatment in the patients with Prostate cancer (CaP) [2-4]. The procedure is used in such patients to relieve urinary retention, though about 50% of patients will pass urine per urethram without catheters after varying lengths of time after hormonal ablation therapy alone[5]. Channeling TURP is associated with complications, which include urinary bladder perforation [6,7]. However, the procedure has become safer over the years in many institutions; hence the complications rates from the procedure have dropped significantly [8,9]. Intestinal obstruction is a very rare complication of TURP as suggested by the scarcity of reports in our search of the medical literatures. We therefore wish to use this case report to highlight the possibility of intestinal obstruction as a secondary complication of urinary bladder perforation.

## Case presentation

A 56-year-old man presented to our unit 6 days after he had a channeling TURP and bilateral orchidectomy performed at another centre. He presented with generalized colicky abdominal pain, abdominal distension and constipation. Though the symptoms started on the first day after the operation, they were not severe and he was discharged from the hospital three days postoperatively. There were associated vomiting, anorexia and hiccoughs, which all started on the fifth day after surgery. The urethral catheter was removed on the third postoperative day and he experienced a significant improvement in his urinary symptoms. He had no known history of hypertension or diabetes patient and there was no previous history of intra-abdominal operations

Clinical examination at presentation revealed mild pallor, fever (T-37.8°C), and bilateral peri-orbital and pedal swellings. The pulses were normal but blood pressure was 170/100 mmHg. The abdomen was distended but soft with no areas of tenderness. Percussion notes were tympanitic and the bowel sounds were hyperactive. Digital rectal examination revealed an enlarged nodular prostate and the rectum contained soft brownish stool. The absent testes and the healing scrotal wound were noted. There was no neuromuscular abnormality.

**Figure 1:**
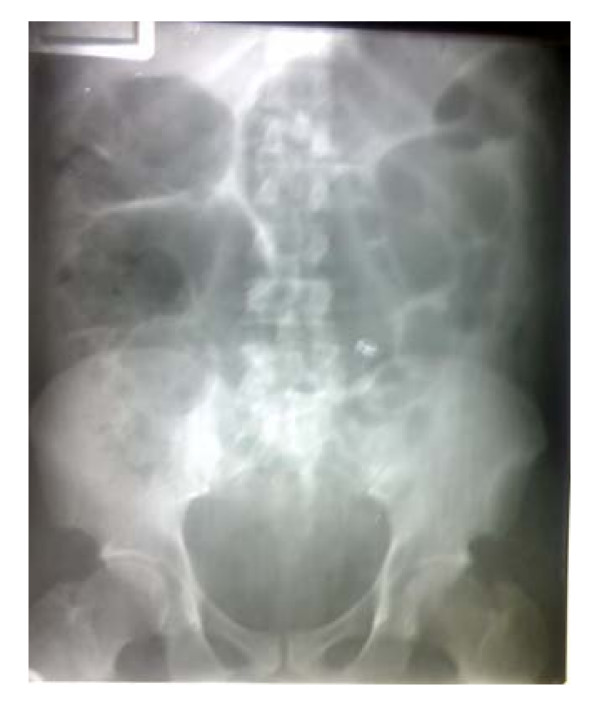
Supine shows distended bowel loops.

**Figure 2:**
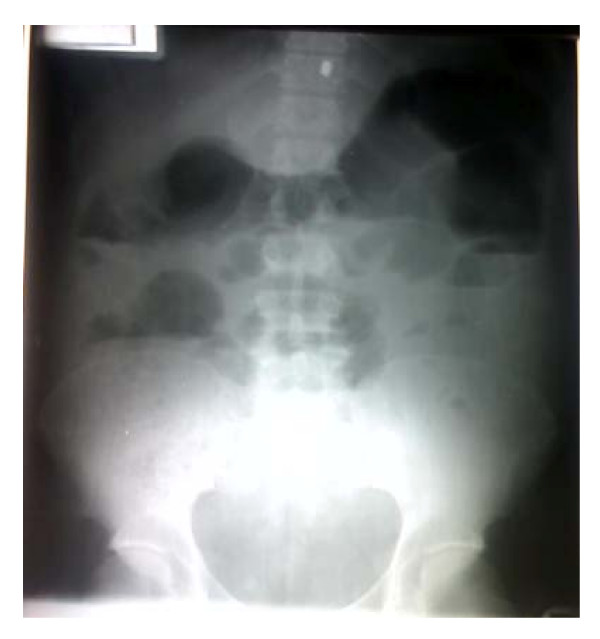
Erect film shows multiple air-fluid levels.

His hemoglobin was 11.2 g/dL; the white cell count was 10.8 × 10^9^/L, (neutrophilia of 73%) and platelet count of 258 × 10^9^/L. He had evidence of renal impairment: serum urea was 28.2 mMol/L (normal range: 2.5–6.5 mMol/L) and serum creatinine level was 744 μmol/L (53–106 μMol/l). He was hyponatraemic (Sodium 126 mMol/L) (135–145 mMol/L) and slightly hyperkalaemic (Potassium, 5.1 mMol/L) (2.9–5.0 mMol/L), Urinary specific gravity was normal. Abdominal radiographs revealed features of intestinal obstruction, with gaseous distension of the bowel especially of the small bowel with multiple air fluid levels and paucity of gas in the pelvis (see Figures 1 and 2). Abdomino-pelvic ultrasound scanning revealed bilateral moderate hydronephrosis, a thickened urinary bladder wall with irregularity in its lower segment and remnants of the prostate gland. Urine microbiology culture yielded growth of *klebsiella pneumoniae*, which was sensitive to ceftazidime and amoxicillin-clavulanic acid combination.

It was apparent that the patient had intestinal obstruction occurring after the Channel TURP with obstructive nephropathy. Initially, conservative management was instituted *vis a vis *nothing by mouth, intravenous fluids (normal saline alternating with 5% dextrose-saline), and intravenous antibiotics using a combination of ceftazidime and metronidazole. The gastro-intestinal tract was decompressed by passing a naso-gastric tube for continuous drainage. A size 18 Fr Foley urethral catheter was passed to monitor the patient's urine output and to decompress the upper urinary tract.

The patient's renal function improved significantly with the conservative management but the abdominal pain and distension persisted and got worse. On the tenth day of admission, he had an exploratory laparotomy. Findings at laparotomy were distended small bowel loops and multiple adhesions involving the dome of the bladder, the small intestine and the large omentum. Adhesiolysis was done and the intestinal loops were released. The postoperative period was uneventful and he was commenced on graded oral diet on the third postoperative day after which he moved his bowel on the same day. The renal status of the patient continued to improve and urethral catheter was removed on the tenth postoperative day, and the patient voided satisfactorily. The serum electrolyte and urea levels at discharge were all within normal ranges (Sodium, 135 mMol/L; Potassium, 4.4 mMol/L; Urea-4.8 mMol/L and creatinine – 88 μMol/L). The patient was discharged from the hospital on antihypertensive medications 22 days after admission. He was seen in the out patient department six months after discharge and his condition had remained satisfactory.

## Discussion

Trans urethral resection of the prostate (TURP) has been recognized as an adjuvant therapy in the management of advanced prostate cancer. This is mainly to create a channel in the obstructive tumour thereby relieving the urinary retention. This may be carried out on the patient *parri passu *with the hormonal ablation therapy to relieve urinary retention. Hormonal ablation alone usually results in the relief of urinary retention in about 50% of patients after varying periods of urethral catheterization. This cannot always be guaranteed. Channeling TURP can be preemptive when it is carried out about the time when the hormonal ablation is initiated as in the case presented. It may also be indicated when the patient's urinary retention is not relieved after a reasonable period after initiating hormonal ablation. Urinary bladder perforation is one of the complications of the procedure [10] and it occurs in less than 1% of cases in some series [8,11]. Most cases of bladder perforation from TURP are managed conservatively by continuous drainage of the urinary bladder, when recognized early. However, some cases of perforation may be missed with no repercussions especially when there are other reasons to keep the urethral catheters on for several days after TURP [11]. Reports of secondary complications resulting from perforation of the urinary bladder have been reported. These are extravesical tumor recurrence [12] intraperitoneal extravasation of irrigating fluid[13] and transurethral resection (TUR) syndrome[14]. The patient in this report sustained an intraperitoneal perforation of the urinary bladder, which was not recognized immediately after the TURP. The greater omentun, in a bid to seal off the perforation on the dome of the bladder, entangled the intestine with the resultant intestinal obstruction. Early recognition and intervention in the management of this complication was responsible for the good outcome in the management of this patient with impaired renal function from the chronic bladder outlet obstruction.

## Conclusion

TURP will remain an important adjunct in the management of patients with advanced CaP. Although, the TURP over the years has become a safe procedure in most experienced hands, possibility of complications occurring should always be borne in mind. Early recognition and management of such complications usually result in good outcome.

## Abbreviations

TURP – Transurethral resection of the prostate; CaP – Cancer of the prostate; Fr – French gauge; mMol/L – Millimole/Litre; μMol/L – Micromole/Litre.

## Competing interests

The author(s) declare that they have no competing interests.

## Authors' contributions

AAP initiated the concept, literature search and write up of the manuscript. KAO involved in case summary. MDA contributed in clinical management of patient and gave approval for final write up. IOO contributed in clinical management of patient and gave approval for final write up. All authors read and approved the final manuscript.

## Consent

Written informed consent was obtained from the patient for publication of this case report and any accompanying images. A copy of the written consent is available for review by the Editor-in-Chief of this journal.
